# Estimating Abundance of Crop‐Foraging Primates in Anthropogenic Landscapes Using Camera Traps

**DOI:** 10.1002/ajp.70087

**Published:** 2025-11-19

**Authors:** Jamie E. T. McKaughan, Philip A. Stephens, Russell A. Hill

**Affiliations:** ^1^ Department of Anthropology Durham University Durham UK; ^2^ Conservation Ecology Group, Department of Biosciences Durham University Durham UK; ^3^ Primate and Predator Project, Alldays Wildlife and Communities Research Centre, Campfornis Game Farm Alldays South Africa; ^4^ SARChI Chair on Biodiversity Value and Change in the Vhembe Biosphere Reserve, Faculty of Science, Engineering and Agriculture University of Venda Thohoyandou South Africa

**Keywords:** chacma baboon, commercial farmland, *Papio ursinus*, REM, South Africa

## Abstract

As human populations grow, competition with wildlife increases, driving more frequent and intense negative interactions between humans and wildlife, of which crop foraging by primates like baboons (*Papio* spp.) is a notable example. Farmers struggle to coexist with baboons, and are particularly concerned by their abundance, with more baboons resulting in more damage. Despite extensive primate research, there are few population estimates, particularly for chacma baboons (*Papio ursinus*). Estimating baboon densities could inform conservation policies, including strategies to mitigate crop losses and negative human‐baboon interactions. Conventional techniques for estimating density are impractical for baboons in commercial farmland or have been unsuccessful in the past, but camera trapping could provide an alternative. Through field tests of the distance sampling with camera traps (CTDS) and the random encounter model (REM) methods, we compare estimates of chacma baboon densities, assessing the potential of these methods for monitoring species that move in large and stable social groups. Chacma baboon density was 1–3 individuals/km^2^, comparable to abundance estimates elsewhere, suggesting that these methods can provide plausible estimates for monitoring baboon populations. Baboon presence and distribution appeared to be more influenced by the availability of sleeping sites than the location of crops, with both seemingly linked to the availability of water. Between‐camera heterogeneity in detections was a large source of imprecision for both methods but further investigation is required to understand how assumptions made in implementing both methods affect accuracy and precision. Future implementations must be refined to successfully identify small changes in long‐term monitoring data.

## Summary

- Crop‐foraging primates are difficult to survey with traditional methods.
- Camera trap survey methods produce useful estimates of chacma baboon abundance in a South African farming landscape.
- These camera trap methods offer potential for monitoring primates in areas of negative human‐wildlife interactions.

## Graphical abstract

Crop‐foraging primates are difficult to survey with traditional methods. Camera trapping offers potential for monitoring primates in areas of negative human‐wildlife interactions.

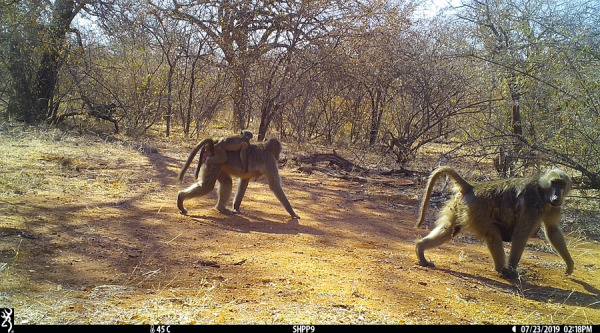

## 1 Introduction

Negative human‐wildlife interactions have increased because of habitat encroachment and resource competition globally (Knight 2001; Herda‐Rapp and Goedeke 2005; Srivathsa et al. 2019). These interactions can have damaging impacts on local communities, ecosystems, and wildlife populations, including human injury or fatality, loss of livelihoods and a decline in biodiversity (Cooper and Ginnett 2000; Nyhus and Tilson 2004; Chase Grey et al. 2017). The increasing overlap between humans and primates in various environments, including natural habitats and human‐modified areas, exacerbates negative interactions between these species (Hill and Webber 2010; Hoffman and O'Riain 2012a; Karimullah et al. 2022). In particular, the transformation of land for agriculture leads to free‐ranging animals moving from natural habitat into agricultural land to crop forage and so conflicts between farmers and wildlife (Findlay and Hill 2020a).

In South Africa, negative human‐wildlife interactions emerge between humans and chacma baboons (*Papio ursinus*) (Hoffman and O'Riain 2012a; Hoffman and O'Riain 2012b; Findlay and Hill 2020a; Findlay and Hill 2020b). Although listed as a species of Least Concern on both the current International Union for Conservation of Nature (IUCN) Red List and the South African Mammal Red List (Hoffman et al. 2016; Sithaldeen 2020), some believe the abundance and range of chacma baboons is much lower than estimated, with suggestions that anthropogenic expansion is causing increasingly fragmented populations (Stone et al. 2012). Chacma baboons are ecologically and behaviorally flexible and inhabit a wide range of habitats in southern Africa (Henzi et al. 2011; Chowdhury et al. 2020). Consequently, they are involved in various negative interactions with humans. In commercial pine plantations, baboons can cause extensive damage to young trees and are shot in considerable numbers (Henzi et al. 2011). In urban settings, such as in the Cape Peninsula, baboons' attraction to human food sources increases their presence in human‐populated areas (Hoffman and O'Riain 2012b). The increased presence of baboons has caused an increase in negative human‐wildlife interactions (Hoffman and O'Riain 2012b) and where more time is spent in these urban areas by baboons, higher numbers of baboon injuries and fatalities occur (Beamish and O'Riain 2014). In our study area ‐ the Alldays area of Limpopo Province ‐ problems between humans and baboons occur most frequently in crop fields (Findlay and Hill 2020a). Foodstuffs cultivated by humans, such as grains, fruit and vegetables, regularly have a higher nutritional value than many natural food sources (Cancelliere et al. 2018), while their location and concentration in crop fields make them an attractive and predictable food source for wildlife. Crop foraging or ‘raiding’ behavior is a major driver of negative human‐primate interactions (Fehlmann et al. 2017; Kaplan et al. 2011), with baboon species (*Papio* spp.) notably prolific (Naughton‐Treves 1997; Hill 2000). Where human livelihoods or food supplies are negatively affected, lethal persecution of baboons is a common response (Marchal and Hill 2009; Findlay 2016).

Many commercial farmers in the Alldays area believe that chacma baboons persist in large populations that are increasing (Findlay 2016). This perception is predominantly based on the frequency of crop foraging events and losses these farmers witness or experience. Arable farming provides baboons with a dense source of nutrients during the dry season when natural food supplies are waning (Findlay and Hill 2020a). This year‐round provision has a two‐fold impact. Firstly, it renders food and water supplies more available year‐round (Findlay 2016), removing constraints on population growth. Baboons respond to greater resource availability very quickly (Bercovitch and Strum 1993), with a potential 20% annual increase in abundance given sufficient resources (Taylor et al. 2016). Secondly, crop foraging animals encounter farmers more regularly, leading to lethal retaliation, often the most common method of baboon control (Findlay and Hill 2020a). The extirpated predator guild (McKaughan et al. 2023) and suspected low leopard population (McKaughan et al. 2024) already reduces natural top‐down population pressure on baboons, further enabling the potential for a large baboon population. Indeed, Taylor et al. (2016) demonstrated that baboon populations can increase five‐fold as apex predators are driven to extinction which, in turn, can lead to a large increase in crop destruction, even in just one event of crop‐foraging (Naughton‐Treves 1997; 1998; Tweheyo et al. 2005).

Management of species that persist outside protected areas relies on accurate information but detailed information on abundance and population trends for chacma baboons is currently lacking (Hoffman et al. 2016). Without this information, it is difficult to formulate future conservation management and policy, including mitigation strategies and potential population control measures such as contraception, that could ultimately help to reduce crop losses and negative human‐baboon interactions. Density estimates that account for seasonal or geographical variations may also help in the development of more targeted coexistence strategies that can be deployed at key times or in key locations (Findlay and Hill 2020b).

Primatologists rarely collect data specifically for density estimation, with observational studies often focusing on group behavior and ranging data (Hill et al. 2003; Hoffman and O'Riain 2012c; Chowdhury et al. 2020). While indirect survey methods have been developed (Plumptre and Reynolds 1996; Kuehl et al. 2007; Cheyne et al. 2008; Gestich et al. 2017), these methods are not appropriate for all primate species and may require additional data that can be expensive or difficult to collect, ultimately limiting the reliability of the density estimates (Cappelle et al. 2019). Most published estimates of baboon density are based upon extrapolating a single or small collection of group counts and their respective ranges (Beamish 2009; Hoffman et al. 2016) collected as a part of behavioral studies. These types of counts are useful, but they are likely imprecise where groups have overlapping home ranges (Slater et al. 2018). The most precise density estimates are achieved through complete counts (Marshall et al. 2008). Some provincial or national estimates of baboons have been made (e.g., Stoltz and Keith 1973; Henzi et al. 2011; Stone et al. 2012), but accurately counting groups is unfeasible on a large scale. In addition, if people regularly threaten animals, accurate counts would likely require habituation that may be impossible and unethical (Fedigan 2010; Green and Gabriel 2020; Hansen et al. 2021; Walton et al. 2021) given it could increase interactions between people and primates. Other methods, such as traditional transect and line sampling methods, as often used on forest primates, can be complicated and produce biased results when employed for large group‐living animals because of the uncertainty of group size or spread (Kiffner et al. 2022; Marshall et al. 2008). They are also difficult to use when flight response to humans are extreme (Bessone et al. 2023), a characteristic of persecuted populations (Bshary 2001). Aerial surveys, while often primarily targeting other species, have successfully provided population data and group counts (Moses et al. 2015; Craig and Gibson 2016; TAWIRI 2019), but aerial surveys are expensive and not always successful for baboons, which can be concealed by thick bush (Leah Findlay, pers. comm.). Efficient monitoring tools are required to estimate populations and trends precisely and accurately, and camera trapping might provide the solution.

Camera traps can provide vital insights to natural behavior without human disturbance or influence (Kelly and Holub 2008). Mark‐recapture methods (Cutler and Swann 1999) have dominated density estimation from camera trap data, but these methods rely on individuals being uniquely recognizable. More recently, models for unmarked individuals have emerged, including Distance Sampling with Camera Traps (CTDS) (Howe et al. 2017) and the Random Encounter Model (REM) (Rowcliffe et al. 2008). The efficacy of these techniques for estimating densities of large group‐living animals has had only limited testing, particularly in‐situ (Pal et al. 2021; Harris et al. 2020; Cappelle et al. 2019; Zero et al. 2013). CTDS is based on point transect sampling, with this established and trusted methodological framework adapted for use with camera trap data. REM is based on an ideal gas model (Rowcliffe et al. 2008), where camera trapping rates are used to model underlying observation processes, with the rate of contact between the observed species and camera traps used to derive a density estimate. In addition to camera parameters, REM also requires animal parameters ‐ speed of movement and group size estimates ‐ to establish a density estimate from the camera trapping rate. Reliable speed of movement data can be difficult and/or expensive to obtain, often requiring extensive additional data collection through field observation or GPS telemetry (Zaumyslova and Bondarchuk 2015). Alternative cheaper methods have been suggested (Rowcliffe et al. 2016), but have not always proven successful (Melville 2019). Reliable speed of movement estimation has perhaps been the greatest constraint on wider use of REM (Palencia et al. 2021b), but because travel data are routinely collected and available in primate studies (Johnson et al. 2015), the potential to estimate terrestrial and semi‐terrestrial primate densities is greatly enhanced (Cappelle et al. 2019).

Here, we report field deployments of the CTDS and REM methods to estimate chacma baboon density in commercial farmland in South Africa, an environment where conventional techniques such as group counts, line transects and aerial counts are impractical or have not produced reliable estimates in the past (Leah Findlay, pers. comm.). Though we could not cross reference estimates with known values, the resulting metrics allow assessments of change in baboon population sizes over time. We aimed to provide these baseline population estimates so that future changes in baboon population abundance could be evaluated and so inform the development of more effective conservation and management strategies. In doing so we assessed the capacity of REM and CTDS for wider use on group‐living species that move in large and stable social groups.

## 2 Methodology

### 2.1 Study Site

**Figure 1:**
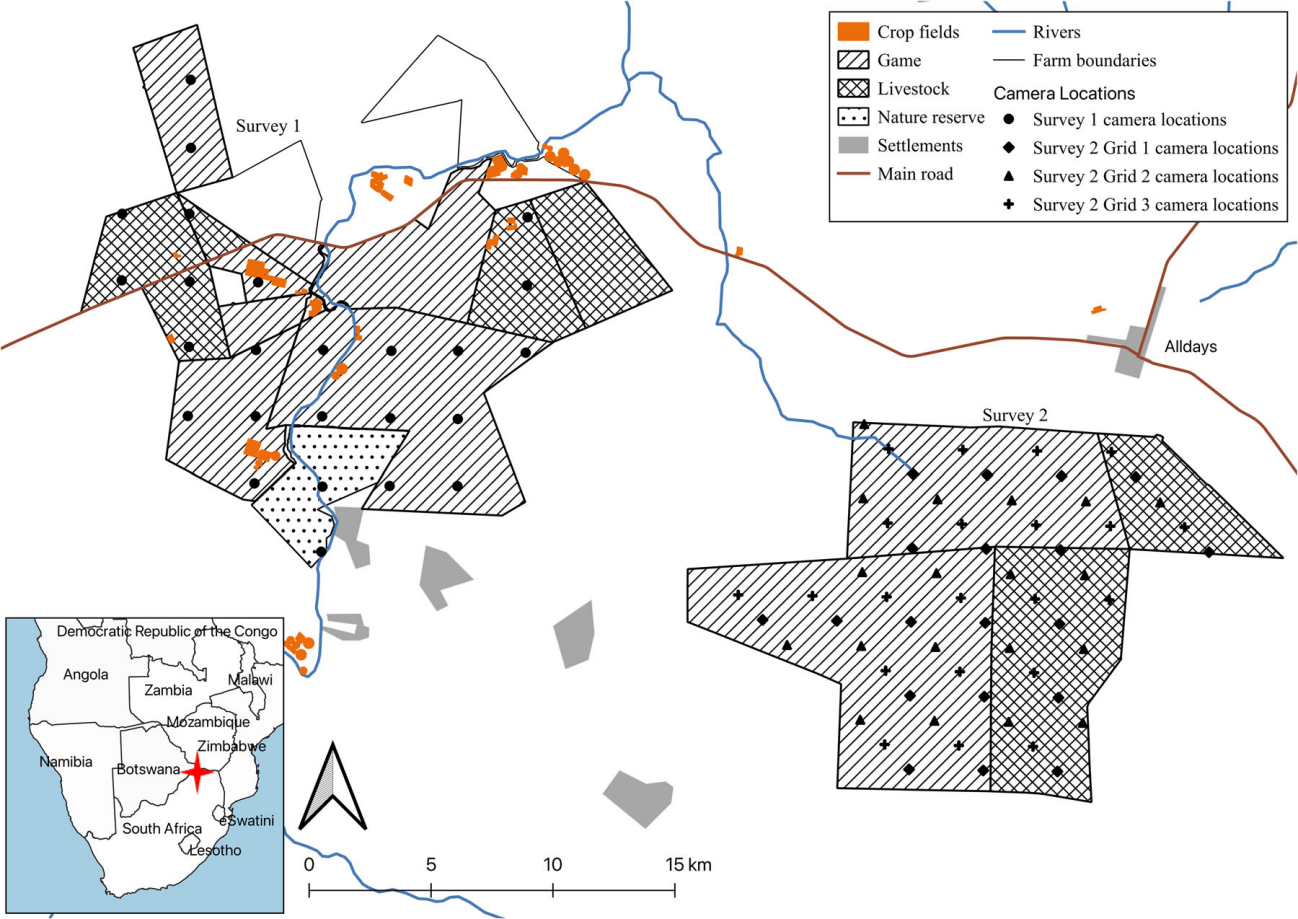
Location of the study area (inset, red cross) and the two camera surveys on farmland in the Alldays area of Limpopo Province, South Africa. The second survey moved cameras south‐east by 1.5 km every 30 days to increase camera locations. Primary economic activities on each property indicated in the key.

We conducted fieldwork in Limpopo Province South Africa, near Alldays in the Blouberg Municipality (central coordinates: −22.674960, 29.020938) (Figure 1). The area has a semi‐arid climate characterized by dry winters (April‐September), with a mean minimum daily temperature of 13°C in June and July and a mean temperature high of 33°C in November. The majority of the mean 650 mm annual rainfall falls in the summer months (October‐March) (Findlay and Hill 2020a). Agriculture is important to much of Limpopo Province's economy (LEDET 2016). Alldays is a mixed‐agricultural area, with large game farms for breeding and hunting, supplemented by livestock farming and crop production, supported by the only perennial river in the area, the Mogalakwena. The river has also supported the growth of large trees that make suitable sleeping sites for baboons (Hoffman and O'Riain 2011). The natural vegetation of the area is predominantly savanna, classified as Limpopo Sweet Bushveld (Mucina and Rutherford 2006), providing baboons with a variety of natural food sources in leaves, fruits, tubers and seeds (Hamilton et al. 1978). Thick acacia bush is prominent throughout the study site. Local farmers grow a diversity of crops (Findlay et al. 2022) and believe these crops attract baboons suggesting greater baboon abundance in areas with greater numbers of fields. All properties were fenced, largely with electrified game fencing (~2.4 m high), although most could be crossed in places by baboons. Some farms had electric fencing around their crops where it can be an effective barrier, dependent upon design (Findlay et al. 2022). Leopards (*Panthera pardus*), were the most significant potential predator of baboons in the area, but estimated numbers were low (McKaughan et al. 2024).

Chacma baboons are group‐living animals, with reported group sizes ranging from 7 to 128 (Beamish 2009; Stone et al. 2012); counts in the Alldays area fall within this range (123: Leah Findlay, unpublished data; 59 and 69: Jamie McKaughan, unpublished data; 25: Rahman (2023)). Group home ranges can overlap (Slater et al. 2018) and differ markedly in size (Hoffman and O'Riain 2012c). Large groups typically occupy larger areas; however, where food resources are abundant, density increases because groups are typically larger with smaller home ranges (Hoffman and O'Riain 2012c).

### 2.2 Field Setup and Data Collection

We conducted two 90‐day camera surveys between June 2019 and March 2020 (Figure 1). Survey Area 1 covered an area of 192 km^2^ that included a mix of game, livestock, crop farms and a nature reserve, while Survey Area 2 covered 200 km^2^ of game and livestock farms only. Area 1 used the same 25 stations for the duration of the study while Area 2 used a migrating camera grid of three 30‐day deployments, moving 1.5 km south‐east with every deployment, totaling 59 locations (McKaughan et al. 2023). We determined camera locations by randomly placing a 3 × 3 km grid over the study areas, setting camera traps (Browning Strike Force HD Pro Model BTC‐5HDP) on the intersections of the grid. We used a GPS device (Garmin GPSmap 60CSx) to locate camera stations in the field and placed camera traps on appropriate trees at a height of 0.7 m and oriented, approximately, toward geographic north. If no suitable tree was available or vegetation blocked the view, we used the nearest alternative location/orientation, ensuring that placement did not increase or decrease detection probability. Orientation varied by ±30° and only one camera was placed further than 30 m from the GPS point, with a relocation distance of 70 m used to avoid placement within an electrified empty boma (enclosure). We used a customized spirit level to align cameras identically relative to ground slope (McKaughan et al. 2023).

To facilitate as near to continuous monitoring as possible (recommended for CTDS: Howe et al. 2017), but without using video settings, we set cameras to the fastest possible recovery time (1 s) between triggers and used ‘6 rapid fire’ burst mode that captures six photographs per trigger, 0.3 s apart (McKaughan et al. 2023). We chose these settings based on pilot data showing that using larger burst modes or video settings would likely fill SD cards before the next available access to some properties hosting camera traps. Despite these settings, our cameras averaged 10.35 s between triggers and 0.49 s between photos in a burst (McKaughan et al. 2023); these figures were accounted for in our analysis (see the Estimating Parameters section below). We serviced cameras approximately every 2 weeks, dependent on property access permissions from landowners, to check the camera remained in its correct position and replace SD cards, and batteries if necessary. We repositioned or replaced moved or broken cameras. In both surveys, one camera was stolen and not replaced.

### 2.3 Distance Sampling With Camera Traps Data Analysis

In CTDS, the potential number of animal recordings by a camera are discretized into snapshot moments for analysis. This method also requires sampling effort to be calculated at each camera location, *k*, with the number of snapshot moments weighted by the detection area covered by the camera. Survey effort is defined as $e_{k}=\frac{{\theta T}_{k}}{2\pi t}$, where *T* is the total sampling time and *t* is the snapshot interval value. In CTDS, density is estimated as:

$$\hat{D}=\frac{\sum_{k=1}^{K}n_{k}}{\pi w^{2}\sum_{k=1}^{K}e_{k}{\hat{P}}_{k}}$$

Where *K* is the set of points, *w* is the truncation distance beyond which any recorded distances are discarded, $n_{k}$ is the number of animal observations at point *k*, with ${\hat{P}}_{k}$ the estimated probability of observing that animal within the detection area and at a snapshot moment.

### 2.4 Random Encounter Model Data Analysis

REM is a model describing rate of contacts between animals and camera traps (encounter rate), while accounting for other elements that influence the trapping rate (Rowcliffe et al. 2008). REM thus uses only the images of individuals considered first contacts; that is, if an animal stays in front of the camera for an extended period of time, only the first image in a sequence is used (Rowcliffe et al. 2008).

$$D=\frac{y}{t}\frac{\pi }{\nu r(2+\theta )}$$

In REM, density is a function of the trapping rate (the number of contacts per unit time, *y*/*t*), animal speed of movement (*v*) and the dimensions of the camera detection zone (calculated from the detection radius, *r* and detection angle, *θ*).

### 2.5 Estimating Parameters

To estimate the area surveyed by the cameras in both methods, we measured both distance to animals and their angle from the center of the viewshed. To avoid bias in encounter rates, individuals that demonstrated a reaction to the camera trap were excluded from analysis (Howe et al. 2017); we identified these cases when an individual clearly changed direction during a burst of images. To measure the distance to animals in photographs, we superimposed a distance overlay grid onto the camera trap images (McKaughan et al. 2023). We tagged distances and angles at the animal's mid‐point using Digikam (version 6.2.0) (Caullier 2019), with 0.5 m measures used up to 10 and 1 m measures up to 25 m. We tagged angles in intervals (0–0.2, 0.2–0.4, 0.4–0.6, 0.6–0.8, 0.8–1.0), where 0 was the center of the photo and 1 the edge, and converted them to absolute angles in R (version 3.6.0) (R Core Team 2013). We downloaded the metadata from the photos, including those with no identified animals, using Exif Tool (version 11.87) (Harvey 2016).

In both REM and CTDS, it is necessary to account for species availability, the proportion of time animals are active and able to be captured by cameras, to avoid over‐ or under‐estimating density (Howe et al. 2017; Palencia et al. 2021b). To estimate the time baboons were active, we used Rowcliffe et al.'s (2014) ‘activity’ package to fit a kernel density distribution to time‐of‐day data in radians, using the ‘fitact’ function in R. We estimated activity for ‘contacts only’ data of baboon groups, defined as images where a baboon group made first contact with the camera. We assumed that a new contact occurred for a group only if there was at least one photo where no baboons were present between images that did contain baboons.

Baboon groups are widely spread and so defining an accurate mid‐point of a group for distance measurements, that is, the central point between all individuals present, is problematic in camera trapping surveys (Marcus Rowcliffe, pers. comm., 2021; Buckland et al. 2001; Palencia et al. 2021b). Accordingly, we tagged baboons as groups for activity analysis and as individual baboons for density analysis in both methods; individual tagging is considered the optimal approach for distance sampling (Howe et al. 2017; Cappelle et al. 2019, 2021) and for REM when no independent estimate of average group size is available (Rowcliffe et al. 2008).

For our CTDS analysis, we used the ‘Trigger adjusted effort’ method from McKaughan et al. (2023) to account for true camera trap performance in survey effort. We removed the true recovery time between triggers, where the camera was inactive (10.35 s), from survey effort (*Tk*) for each trigger event, and because the measured time between snapshots within bursts was 0.49 s, snapshot intervals were set at t=0.5 s. We calculated detection area from the estimated detection radius and effective angle of detection, θ, which was calculated for each survey using the absolute angles of the baboons to produce a detection function. This approach accounts for changes in detection probability with distance from the camera, or towards the periphery of the field of view; both of these will affect the effective detection area.

We used the ‘Distance’ package (Miller et al. 2019) in R to estimate density, following Buckland et al. (2001) and Howe et al. (2017) closely. Models considered for analysis were: half‐normal, with 0 and 1 Hermite polynomial adjustment terms; uniform with 1 and 2 cosine adjustment terms; and hazard rate with 0, 1, and 2 cosine adjustment terms. We used QAIC to choose between candidate models (Howe et al. 2019) to account for the potential overdispersion introduced through violation of the assumption that observations are independent events (Buckland et al. 2001). We left‐truncated data where fewer than anticipated observations occurred nearer the camera traps (Buckland et al. 2001). We right‐truncated when detection probability was lower than 0.15 (Buckland et al. 2001). We estimated variance using a nonparametric bootstrap, resampling points with replacement 1000 times from the distribution of underlying parameters (detection function, encounter rate and activity level).

For our REM analysis, we also tagged individuals with a contact tag when they appeared for the first time in an image and could have credibly triggered the camera (i.e. within 25 m of the camera), regardless of whether other group members were already present. These individuals were then not ‘contacts’ for the duration of their stay in the FOV. They were considered a new contact if they left the FOV and re‐entered (within 25 m) (Rowcliffe et al. 2008), as it is impossible to know if the individual re‐appearing is the same individual.

We applied distance sampling analysis to the recorded animal positions for those tagged as contacts (Marcus Rowcliffe et al. 2011) to estimate the camera detection zone, providing estimates of *r* and θ, along with associated variance in these parameters. The total sampling time of each camera location was matched with that used for the Distance Sampling with Camera Traps to account for the camera trap performance. For the speed of animal movement (*v*) and the trapping rate parameters (*y*/*t*) we used day range, which was estimated using the sum of the product of the baboon's speed while active and the proportion of the activity level associated with movement. Activity level and its variance were estimated as described for CTDS, to ensure that uncertainty in this parameter affected density estimates obtained by both methods, equally. We estimated speed while active from collar data taken in 2013–14 from a female inhabiting part of our first survey area (Walton et al. 2021). We calculated the speed while active using the *linear_speed* function from the swaRm package in R (Garnier 2021), using the speed of all straight‐line movements between each GPS fix. We estimated the variance in speed while active from the variation in speeds of each movement between fixes. The collar took GPS fixes between 05:00 and 19:00 South African Standard Time at hourly intervals, with a further fix at 24:00 to identify sleeping sites (*N* = 5,728 fixes) (Walton et al. 2021). There were no unsuccessful GPS fixes and mean time to GPS fix was 28 s (Walton et al. 2021). We used information from McCann et al. (2021) to calculate how speed while active and density estimates would be affected by varying GPS fix times.

We estimated trapping rate variance using nonparametric bootstrapping, resampling camera locations with replacement. We incorporated the independent variance estimates of each parameter (*v*, *r*, θ and activity), using the delta method (Seber 1982, as cited in Rowcliffe et al. 2008) to compute an overall variance estimate for density.

## 3 Results

**Figure 2:**
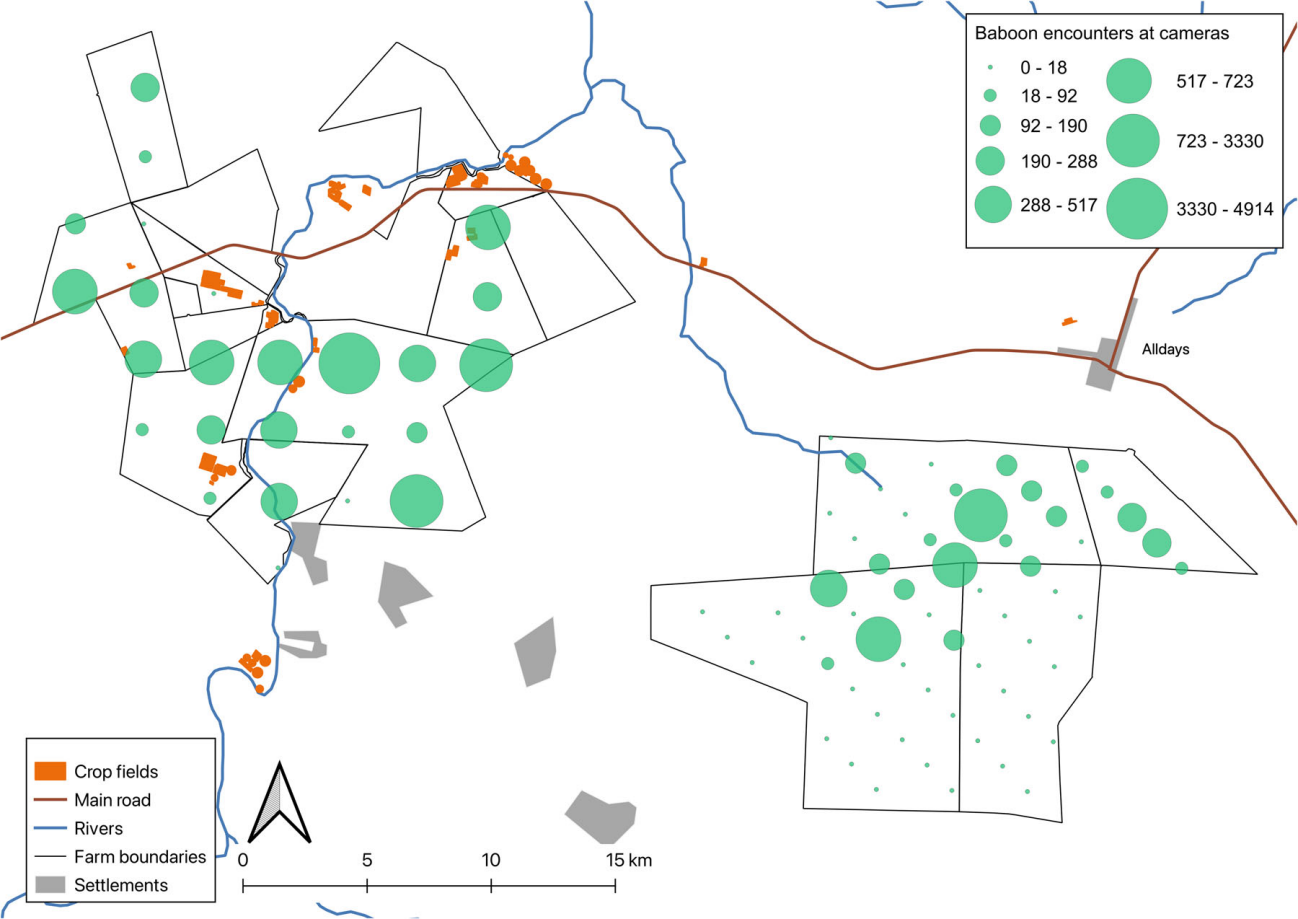
Locations of crop farms in the Alldays area with the total number of observations per camera trap of chacma baboons across the two survey areas.

We obtained 72,360 photos of animals from 25 camera placements in Area 1 (mean 2,894.4, range 549 – 14,336 per location) and 46,870 animal images across 59 locations in Area 2 (mean 794.4, range 62 – 6,930 per location). Survey effort totaled 110,023 h. A total of 20,335 individual chacma baboon observations were obtained across the two survey periods (Area 1: 15,170; Area 2: 5,165), with 84% (k = 21) and 44% (k = 26) of camera locations having at least one observation for Area 1 and 2, respectively (Figure 2). The effective detection distances between methods were similar for each Area, but differed considerably between the two Survey Areas (Table 1). Baboon activity patterns were very similar for the two areas (Table 1).

**Table 1:** Summary of survey parameters and density estimates of chacma baboons estimated with CTDS and REM at the two survey sites.

Method	Parameters	Survey area 1	Survey area 2
	Activity level	0.26 SE ± 0.034	0.33 SE ± 0.048
	Sites captured	21/25	26/59
CTDS	Observations	12554	4827
	Effective detection distance (m)	11.7 SE ± 0.13	7.8 SE ± 0.23
	Truncation distances	2 ‐ 24	1.5 ‐ 24
	Effective detection angle (Radians)	0.7 SE ± 0.0064	0.62 SE ± 0.009
	Encounter rate	0.00032	0.00019
	Probability of detection	0.24	0.11
	**Density/km** ^**2**^ **[95% CI Intervals]**	**2.82 [1.4–5.48]**	**2.97 [0.44–21]**
	CV	4.37	211.09
REM	Observations (contacts)	1311	537
	Effective detection distance (m)	12.33 SE ± 0.33	7.21 SE ± 0.44
	Effective detection angle (Radians)	0.74 SE ± 0.004	0.74 SE ± 0.006
	Speed (m/s)	1.38SE ± 0.027	1.38SE ± 0.027
	Trapping rate	0.63	0.34
	**Density/km** ^**2**^ **[95% CI Intervals]**	**1.28 [0.63–2.28]**	**0.94 [0.5–1.47]**
	CV	0.34	0.27

QAIC scores showed the Hazard rate model with no adjustment terms best fitted the CTDS data. This yielded density estimates of 2.82 and 2.97 baboons/km^2^ for Area 1 and 2, respectively, but the coefficient of variation (CV) from the two surveys differed markedly (4.37 vs. 211.09) (Table 1).

Daily travel distance was estimated at 4.9 km (Walton et al. 2021) and thus travel was estimated as 1.38 m/s ± 0.027 (SE). REM densities were estimated at 1.28 and 0.94 baboons/km^2^ for Area 1 and 2, respectively, with similar CVs of 0.34 and 0.27 respectively. As simulated GPS fix times become more frequent, daily travel speed increases, resulting in lower density estimates (Table 2).

**Table 2:** REM density estimates for chacma baboons based on predicted changes in speed of movement estimates from different GPS fix regimes. Fix time: GPS fix interval; DPL: Daily Path Length (m); DPL % change: percentage change in predicted DPL compared to 1 h fixes from Walton et al. (2021); Speed: Straight line speed while active estimate (m/s); S1 $\hat{D}$, S2 $\hat{D:}$ Survey area 1 and survey area 2 density estimates, respectively, computed using the relevant Speed.

Fix time	1 s	1 s smoothed	30 s	60 s	5 min	15 min	1 h	2 h
**DPL**	10860	9810	7303	6583	5246	4336	3588	2713
**DPL % change**	202.7%	173.4%	103.5%	83.5%	46.2%	20.8%	—	−24.4%
**Speed**	4.170	3.770	2.800	2.530	2.020	1.670	1.380	1.040
**S1** $\hat{D}$	0.422	0.467	0.629	0.696	0.872	1.050	1.280	1.690
**S2** $\hat{D}$	0.309	0.342	0.461	0.510	0.639	0.772	0.940	1.240

## 4 Discussion

We estimated densities of chacma baboons across two survey areas in a mixed farming landscape in South Africa using camera trap distance sampling (CTDS) and the random encounter model (REM). The densities varied between locations and methodologies, but collectively suggested chacma baboon density was 1–3 individuals/km^2^. The REM analysis produced lower and more precise estimates in both survey areas than CTDS. Importantly, however, we suggest that camera trapping methods can produce meaningful density estimates for baboons in environments where more traditional measures are not feasible. Beyond discussing the abundance estimates in the context of our study site, two main points emerge: the effectiveness of camera traps compared to conventional methods for primate populations, and the methodological nuances inherent in estimating density with camera traps for species with large and stable social groups in human dominated landscapes.

### 4.1 Abundance Estimates

Although there are few studies, our results fall within the range of previous chacma baboon density estimates from across southern Africa (e.g., Stoltz and Keith 1973; Stone et al. 2012). Chacma baboon group count and home range data from 23 groups have a mean density of 5.1 baboons/km^2^, ranging from 0.48 to 23.9 baboons/km^2^ (Table S1). Our estimates are at the lower end of this range, however, this density comparison can be misleading as it ignores potential range overlap between groups, and it also represents only areas known to be used by baboons (whereas camera trap surveys may extend beyond baboon ranges); group count/home range density estimates will be elevated as a result. More comparable data from Hoffman and O'Riain (2012c), based on multiple group counts and information on a wider survey area, still place our estimates at the lower end of their range for individual groups (1.3–12.1 individuals/km^2^) but, crucially, indicate that our estimates are in line with their global estimate based on the range area (c.250 km) (Table 3). These results suggest that camera traps can produce meaningful population density estimates but highlight that while abundance might be similar across large areas, the micro‐level distribution and density can vary greatly. Camera trap studies in areas with known densities, like the Cape Peninsula, would help provide a clearer understanding of comparative abundance and accuracy in camera trapping and would offer context for establishing typical density levels across the chacma baboon range.

**Table 3:** Chacma baboon densities estimated from group size and home range data from Hoffman and O'Riain (2012c). Group counts are precise group numbers, while home ranges are calculated using the quadrat method.

Group name	Group size	Home range (km^2^)	Density (/km^2^)
KK	49	37.65	1.3
SWB	26	9.26	2.8
BB	16	5.63	2.8
SK	24	8.28	2.9
CP	22	7.46	2.9
DG	35	10.58	3.3
PR	36	9.05	4
RH	16	1.54	10.4
TK	115	9.5	12.1
All groups	339	250^a^	1.3
All Cape Peninsula	339	470	0.7

a Approximate area in which all groups were collectively ranging, as reported by Hoffman and O'Riain (2012c), which is not the total of each group's home range.

While our results indicate similar baboon abundance to other studies in human‐modified habitat, the distribution of baboons and their local densities within a larger area is an important factor in human‐baboon interactions. Local density can influence people's perception of how many baboons occupy a wider area and the approaches they take to protect their livelihoods. Farmers in the Alldays community have repeatedly emphasized that baboons had a high population density, especially around crop farms (Findlay 2016). Baboons were detected more frequently across all cameras in Area 1 (84%) (Figure 2), which had more crop fields and larger trees along the Mogalakwena River that provided sleeping sites, a significant factor in their ranging behavior (Hoffman and O'Riain 2011). Area 2 yielded fewer observations, with captures concentrated in the central north and north‐east region (Figure 2). Cameras in areas with thick acacia bush and few large trees captured fewer or no baboons compared to the central and northern regions, where there are more large trees and *Grewia* spp. that provide abundant sleeping sites and food supplies. As the two survey areas had similar climatic conditions and predator populations (McKaughan et al. 2023), and supplementary food provided for game and livestock in the dry season was available in both survey areas, these distribution patterns suggest that the availability of sleeping sites, rather than the location of crops, may influence the presence of baboon groups. Nevertheless, the occurrence of tall riverine vegetation indicates the presence of sufficient water for crop cultivation, and so the two are linked. In any event, the distribution and regularity of baboon observations in Area 1 may lead to the impression of inflated abundance by farmers, despite no actual difference in abundance between the surveyed areas. This false perception emphasizes the importance of representative population estimates for larger areas than a single baboon group range.

### 4.2 Methodological Considerations for Group‐Living Species

Density estimates were more precise for REM than CTDS. The variability in encounter rates resulting from habitat use was a leading factor contributing to the imprecision of density estimates from CTDS, where low activity behaviors in front of a camera have a large effect compared to no effect on REM estimates. Precise estimates are critical if they are to be used to monitor change over time. Compared to other species, the group‐living nature of baboons may exacerbate the impact of varied encounter rates on precision, as there could be multiple individuals staying in the field of view for repeated events (Pal et al. 2021), creating greater extremes of observations. Both survey areas had a small number of camera stations that captured far more baboons than other stations, which, in conjunction with other camera locations capturing no baboons, introduces greater uncertainty when bootstrapping. As such, estimating density with CTDS may be challenging for species like baboons, particularly when they are patchily distributed in the landscape.

Other studies on group living species analyzing individuals as the unit of observation using CTDS have achieved more precise density estimates that are comparable to known counts (Cappelle et al. 2019). However, the behavior of some social species may mean they are less prone to variability in encounter rates, particularly if surveys are restricted to a known range. For example, the fission fusion social organization of Western chimpanzees (*Pan troglodytes*), where communities are territorial and animals forage alone or in small subgroups, contrasts with the large, stable groups of chacma baboons of 50–100 individuals that move and forage as a cohesive unit with considerable range overlap between groups. This results in baboon groups being clustered on the landscape, which can generate greater variability among camera trap locations, while chimpanzees are more evenly distributed. Furthermore, in areas of crop farming, chacma baboons use a “sit‐and‐wait strategy”, where they engage in low activity behaviors for an extended period of time before using high activity bursts to forage on crops (Walton et al. 2021). Where these low activity behaviors occur in front of a camera, impacts variability among camera locations. Even species with smaller group sizes, such as bharal (*Pseudois nayaur*), considerable imprecision in initial density estimates was attributed to one particular location (Pal et al. 2021).

Due to the random placement of cameras for these methods, there is no way to mitigate these types of problems. Although Cusack et al. (2015) and Pal et al. (2021) addressed the imprecision by removing certain locations from their data analysis, this might bias estimates, and it is well established that animals will utilize some areas more heavily than others (e.g., carnivores on roads (Swanepoel et al. 2016); primates near sleeping sites (Hoffman and O'Riain 2011)). Migrating the camera grid in Area 2, with the aim of increasing survey locations to improve precision, did not improve precision of the CTDS estimates, as seen with other species (McKaughan et al. 2023). Nevertheless, significantly increasing number of sampling sites may reduce variance (Capelle et al. 2021). Interestingly, the REM estimates from Area 2 were slightly more precise, despite the area having greater variation in encounter rate among locations, smaller sample size, and reduced detectability. The increased camera locations in Area 2 may thus have had more of an influence on the REM results. While the estimates from REM will also have many survey locations with zero baboon observations, the upper extremes of detections were much lower because using only first contacts avoids accumulation of repeated measures. For CTDS, using smaller values of *t* increases the rate at which observations accumulate and so differences in the numbers of location‐specific observations for CTDS relative to REM are also greater. While other studies have used larger values of *t* in such instances (Palencia et al. 2021a), our value of *t* was defined by camera performance. Further research is needed to determine how the selection of *t* influences precision.

Decisions about truncation may also influence precision. In CTDS left truncating data is considered appropriate when the distribution reflects missed detections close to the point, but it can cause positive bias in estimates if the cause is just mild avoidance of the camera (Buckland et al. 2001). If we did not left‐truncate our data, the densities were only marginally impacted, but the precision improved markedly (see Supplementary Information, Figure S1 and Figure S2). In our study, the baboons often interacted with the cameras rather than avoided them, but in removing these interactions to prevent them biasing the estimates, it is then difficult to conclude whether the resulting lack of observations near the point was truly reflective of the population or whether observations were still missed (e.g., young baboons moving under the camera traps). Further research is needed to determine when left truncation is warranted.

Despite the potential value of camera traps for density estimation, careful methodological considerations are thus still required. Defining when an individual of a group‐living species is a new contact, as is required for REM, is difficult from camera trap images for several reasons. Firstly, when multiple individuals are captured in one trigger of the camera it is impossible to know which animal triggered the camera. Secondly, when an individual leaves the field of view and returns again, it can be difficult to know if this is a new individual or one already captured. Finally, where the true camera trap performance does not reflect the camera settings, as was the case here, some contacts might be missed during the cameras extended recovery times as an animal might enter and then leave the FOV before the camera is active again. We set several criteria to classify when an animal was considered a new contact, but this requires further research to understand the effects of certain assumptions on density estimates and to find the optimal strategy to consistently classify contacts of group‐living animals.

The speed of movement parameter is also fundamental to REM, but can be difficult to estimate accurately. We used data from just a single collar, and there are questions relating to the suitability of collar data with longer fix times because they likely underestimate distance travelled (McCann et al. 2021); we showed that different fix times can have considerable impact on density estimates using REM with, for example, an approximately 50% increase in density from 1 h fixes compared to 5 min fixes. Further investigation into the optimal granularity of GPS fixes to use when estimating density with REM is integral to building confidence in using telemetry data in density estimates from REM going forwards. Furthermore, if collaring individuals to obtain GPS data, then using capture‐recapture methods to estimate density may be more appropriate as animals can be uniquely marked during the capture process (Gilbert et al. 2021). Movement speed has also been estimated from camera trap data as an alternative to GPS collars or follows, which would be particularly useful in areas where negative human‐wildlife interactions are high. Having previously had its applicability questioned (Melville 2019; Melville and Strauss 2022), the reliability of speed estimated from camera trap data as a viable alternative has improved (Palencia et al. 2019; Palencia et al. 2021a) producing results for several species (Pfeffer et al. 2018; Kavčić et al. 2021; Palencia et al. 2022), and as such warrants further research, particularly with group‐living species.

## 5 Conclusion

Density estimation of baboons in areas of human‐baboon coexistence is a challenging task for practical and ethical reasons. Our results demonstrate the promise of camera traps as a suitable and effective method for monitoring baboon densities in areas of human‐baboon coexistence. They are less labor intensive and more efficient than traditional line transects (Cappelle et al. 2019) and are also less obtrusive. Although the first outlay of camera traps is typically expensive, they have multiple applications beyond just density estimation, while with technological advances, such as AI‐based photo classifications (Vélez et al. 2023; Fergus et al. 2024), automated distance measurement (Ternyik et al. 2024) and solar cameras (Braczkowski 2023), the time and cost involved in camera trapping are expected to decrease. Camera traps thus offer a cost‐effective alternative to the labor‐intensive fieldwork required for long‐term monitoring (Cappelle et al. 2019). Nevertheless, careful planning of camera trap surveys would be needed to ensure that robust density estimates are generated that can inform monitoring of population trends and conservation and management strategies.

## Author Contributions

**Jamie E. T. McKaughan:** conceptualization (equal), formal analysis (lead), methodology (lead), writing – original draft (lead), writing – review and editing (lead). **Philip A. Stephens:** conceptualization (equal), supervision (equal), writing – review and editing (equal). **Russell A. Hill:** conceptualization (equal), supervision (equal), writing – review and editing (equal).

## Conflicts of Interest

The authors declare no conflicts of interest.

## Supporting information

SUPPLEMENTARY INFORMATION.

## Data Availability

The data used in this article is openly available on OSF (McKaughan et al. 2025).
