# Supplementary material for: Estimating Abundance of Crop‐Foraging Primates in Anthropogenic Landscapes Using Camera Traps
Source: Am J Primatol. 2025 Nov 19;87(11):e70087. doi: 10.1002/ajp.70087 (PMC12631099; doi:10.1002/ajp.70087)
Supplement: Supplementary file 1 — SUPPLEMENTARY INFORMATION. [file AJP-87-e70087-s001.docx]

**Estimating abundance of crop-foraging primates in anthropogenic landscapes using camera traps**

**Supplementary information**

Analytical CVs of original analysis

Survey Area 1:

Density 2.82 (CV 0.453)

Survey Area 2:

Density 2.97 (CV 0.442)

No left-truncation

Survey Area 1:

Density 2.6 (95% CIs 1.1 - 6.2) (SE $\pm$1.15) CV 0.32 (Analytical CV 0.452)

Survey Area 2:

Density 2.6 (95% CIs 1.1 – 5.9) (SE $\pm$1.12) CV 1.37 (Analytical CV 0.436)


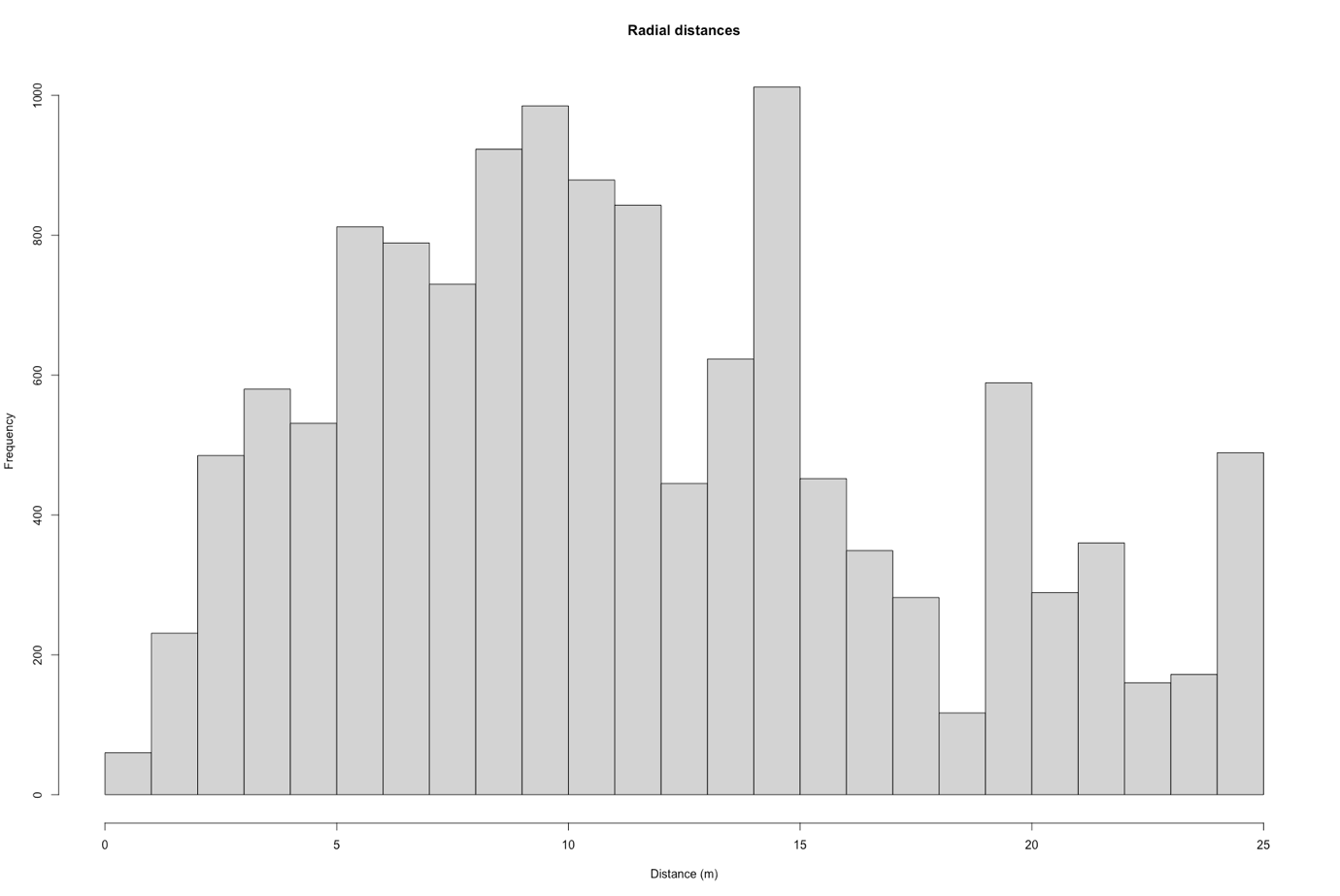


Figure S1. Frequency of radial distances of chacma baboon observations at 1 m intervals from Area 1.


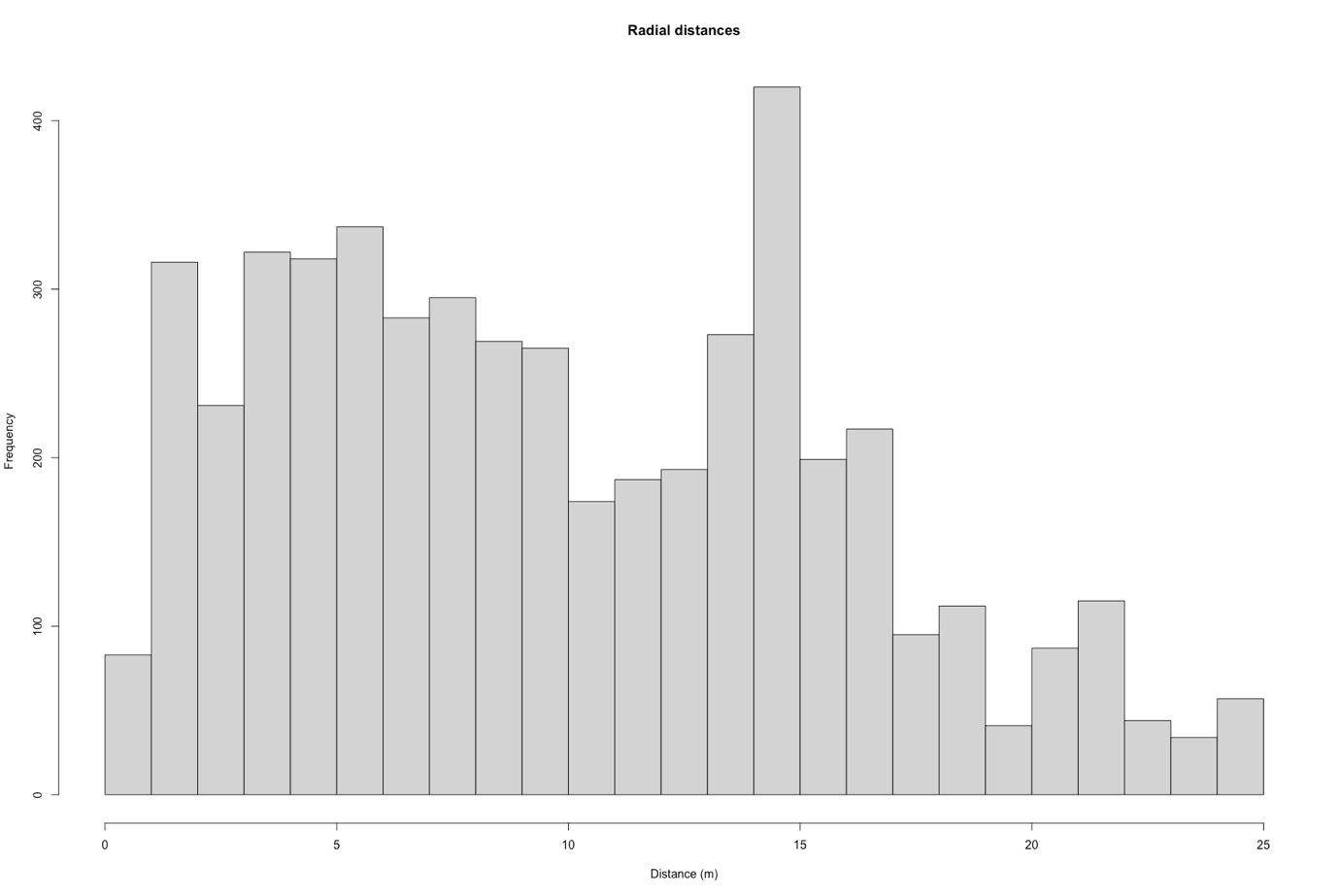


Figure S2. Frequency of radial distances of chacma baboon observations at 1 m intervals from Area 2.

Table S1. Chacma baboon densities in southern Africa estimated from group size and home range data, ordered by density. Group counts are precise group numbers, while home ranges are calculated using minimum convex polygons, kernel density estimates or the quadrat method.

| **Study site (group name), Country** | **Group Size** | **Home Range (km^2^)** | **Density (/km^2^)** | **References** |
| --- | --- | --- | --- | --- |
| Drakensberg (High), SA | 9 | 18.9 | 0.48 | Whiten et al. 1987 |
| Tsaobis (L troop), Namibia | 32 | 26.75 | 1.2 | King 2008 |
| Cape Peninsula (Kanonkop), SA | 56 | 45.3 | 1.24 | Hoffman 2011; Lewis and O’Riain 2017 |
| Drakensberg (Low), SA | 14 | 10 | 1.4 | Whiten et al. 1987 |
| Suikerbosrand, SA | 38 | 26.7 | 1.42 | Segal 2008; Slater et al. 2018 |
| De Hoop (Small troop), SA* | 19 | 12.36 | 1.53 | Hill 1999 |
| Blyde Canyon (Bourke’s Luck), SA | 18 | 10.35 | 1.74 | Marais et al. 2006 |
| De Hoop (Vlei troop), SA* | 42 | 18.76 | 2.24 | Hill 1999 |
| Cape Peninsula (O troop), SA | 85 | 37 | 2.3 | Davidge 1978b; Davidge 1978a |
| Honnet (RB troop), SA* | 59 | 23.31 | 2.53 | Stoltz and Saayman 1970 |
| Drakensberg (London), SA | 42 | 14.96 | 2.81 | Henzi et al. 2011 |
| Drakensberg (Driekop), SA | 40 | 14.13 | 2.83 | Henzi et al. 2011 |
| Cape Peninsula (Plauteau Road), SA | 33 | 9.05 | 3.65 | Hoffman and O’Riain 2012c; van Doorn et al. 2010 |
| Suikerbosrand (Kraal), SA | 88 | 22.38 | 3.93 | Anderson 1981 |
| Tsaobis (J troop), Namibia | 57 | 12.25 | 4.65 | King 2008 |
| Mkuzi game reserve (Flash’s), SA | 28 | 4.72 | 5.93 | Gaynor 1994 |
| Mkuzi game reserve (Mtshopi), SA | 71 | 11.76 | 6.04 | Gaynor 1994 |
| Mkuzi game reserve (Darth’s), SA | 43 | 6.88 | 6.25 | Gaynor 1994 |
| Alldays, SA* | 22 | 2.95 | 7.46 | Rahman 2023 |
| Wildcliff, SA | 115 | 11.98 | 9.6 | Pebsworth et al. 2012 |
| Cape Peninsula (RH troop), SA | 16 | 1.5 | 10.67 | Hoffman 2011; Hoffman and O’Riain 2012c; Kaplan et al. 2011 |
| Cape Peninsula (Tokai), SA | 115 | 9.5 | 12.11 | Hoffman and O’Riain 2011 |
| Cape Peninsula (MT1 troop), SA | 65 | 2.72 | 23.9 | Dubay 2018 |
| * Density estimates were calculated from the mean group count for the respective group in each study | | | | |

References

Anderson, C. M. (1981). Intertroop relations of chacma baboon (*Papio ursinus*). *International Journal of Primatology* 2(4), 285-310.

Davidge, C. (1978a). Activity patterns of chacma baboons (*Papio ursinus*) at Cape Point. *Zoologica Africana* 13(1), 143-155. Doi: 10.1080/00445096.1978.11447612

Davidge, C. (1978b). Ecology of baboons (*Papio ursinus*) at Cape Point. *Zoologica Africana* 13(2), 329-350. doi: 10.1080/00445096.1978.11447633

Dubay, S. (2018). Behavioural and physiological responses of chacma baboons (Papio ursinus) to wildfire in the Cape Peninsula of South Africa. [Unpublished doctoral dissertation]. University of Cape Town.

Gaynor, D. (1994). Foraging and feeding behaviour of chacma baboons in a woodland habitat [Unpublished doctoral dissertation]. University of Natal.

Henzi, S. P., Brown, L. R., Barrett, L., & Marais, A. J. (2011). Troop size, habitat use, and diet of chacma baboons (*Papio hamadryas ursinus*) in commercial pine plantations: implications for management. *International Journal of Primatology*, *32*(4), 1020–1032. https://doi.org/10.1007/s10764-011-9519-6

Hill, R. A. (1999). Ecological and demographic determinants of time budgets in baboons: implications for cross-populational models of baboon socioecology [Unpublished doctoral dissertation]. University of Liverpool.

Hoffman, T. (2011). The spatial ecology of chacma baboons (*Papio ursinus*) in the Cape Peninusula, South Africa- towards improved management and conservation strategies [Unpublished doctoral dissertation]. University of Cape Town.

Hoffman, T. S., & O’Riain, M. J. (2012c). Troop size and human-modiﬁed habitat affect the ranging patterns of a chacma baboon population in the Cape Peninsula, South Africa. *American Journal of Primatology*, *74*, 853–863. https://doi.org/DOI 10.1002/ajp.22040

Kaplan, B. S., O’Riain, M. J., van Eeden, R., & King, A. J. (2011). A low-cost manipulation of food resources reduces spatial overlap between baboons (*Papio ursinus*) and humans in conflict. *International Journal of Primatology*, *32*(6), 1397–1412. https://doi.org/10.1007/s10764-011-9541-8

King, A. J. (2008). Leadership, coordinated behaviour, and information use in a social primate [Unpublished doctoral dissertation]. University College London.

Lewis, M. C., & O’Riain, M. J. (2017). Foraging profile, activity budget and spatial ecology of exclusively natural-foraging chacma baboons (*Papio ursinus*) on the Cape Peninsula, South Africa. *International Journal of Primatology*, *38*(4), 751–779. <https://doi.org/10.1007/s10764-017-9978-5>

Marais, A. J., Brown, L. R., Barrett, L. & Henzi, S. P. (2006). Population structure and habitat use of baboons (*Papio hamadryas ursinus*) in the Blyde Canyon Nature Reserve. *Koedoe* 49(2), pp. 67-76. doi: 10.4102/koedoe.v49i2.117.

Pebsworth, P. A., MacIntosh, A. J. J., Morgan, H. R. & Huffman, M. A. (2012). Factors influencing the ranging behavior of chacma baboons (*Papio hamdryas ursinus*) living in a human-modified habitat. *International Journal of Primatology* 33(4), 872-887. doi: 10.1007/s10764-012-9620-5.

Rahman, M. M. (2023). Behavioural and physiological responses to anthropogenic food sources in chacma baboons [Unpublished doctoral dissertation]. Durham University.

Segal, C. (2008). Foraging behaviour and diet in chacma baboons in Suikerbosrand Nature Reserve [Unpublished doctoral dissertation]. University of the Witwaterstrand.

Slater, K., Barrett, A., & Brown, L. R. (2018). Home range utilization by chacma baboon (*Papio ursinus*) troops on Suikerbosrand Nature Reserve, South Africa. *PLoS ONE*, *13*(3). https://doi.org/10.1371/journal.pone.0194717

Stoltz, L. P. & Saayman, G. S. (1970). Ecology and behaviour of baboons in the Northern Transvaal. *Annals of the Transvaal Museum* 26(5), 99-143.

Van Doorn, A. C., O’Riain, M. J. & Swedell, L. (2010). The effects of extreme seasonality of climate and day length on the activity budget and diet of semi-commensal chacma baboons (*Papio ursinus*) in the Cape Peninsula of South Africa. *American Journal of Primatology* 72, 104-112. doi: 10.1002/ajp.20759.

Whiten, A., Byrne, R. W., & Henzi, S. P. (1987). The behavioral ecology of mountain baboons. In *International Journal of Primatology,* *8*(4).
